# Machine Learning to Predict Contrast-Induced Acute Kidney Injury in Patients With Acute Myocardial Infarction

**DOI:** 10.3389/fmed.2020.592007

**Published:** 2020-11-13

**Authors:** Ling Sun, Wenwu Zhu, Xin Chen, Jianguang Jiang, Yuan Ji, Nan Liu, Yajing Xu, Yi Zhuang, Zhiqin Sun, Qingjie Wang, Fengxiang Zhang

**Affiliations:** ^1^Department of Cardiology, The Affiliated Changzhou No. 2 People's Hospital of Nanjing Medical University, Changzhou, China; ^2^Section of Pacing and Electrophysiology, Division of Cardiology, The First Affiliated Hospital of Nanjing Medical University, Nanjing, China; ^3^Department of DSA, The Affiliated Changzhou No. 2 People's Hospital of Nanjing Medical University, Changzhou, China; ^4^School of Clinical Medicine, The Affiliated Changzhou No. 2 People's Hospital of Nanjing Medical University, Changzhou, China

**Keywords:** machine learning, Random Forest algorithm, logistic regression, predictive models, contrast induced acute kidney injury, acute myocardial infarction

## Abstract

**Objective:** To develop predictive models for contrast induced acute kidney injury (CI-AKI) among acute myocardial infarction (AMI) patients treated invasively.

**Methods:** Patients with AMI who underwent angiography therapy were enrolled and randomly divided into training cohort (75%) and validation cohort (25%). Machine learning algorithms were used to construct predictive models for CI-AKI. The predictive models were tested in a validation cohort.

**Results:** A total of 1,495 patients with AMI were included. Of all the patients, 226 (15.1%) cases developed CI-AKI. In the validation cohort, Random Forest (RF) model with top 15 variables reached an area under the curve (AUC) of 0.82 (95% CI: 0.76–0.87), while the best logistic model had an AUC of 0.69 (95% CI: 0.62–0.76). ACEF (age, creatinine, and ejection fraction) model reached an AUC of 0.62 (95% CI: 0.53–0.71). RF model with top 15 variables achieved a high recall rate of 71.9% and an accuracy of 73.5% in the validation group. Random Forest model significantly outperformed logistic regression in every comparison.

**Conclusions:** Machine learning algorithms especially Random Forest algorithm improves the accuracy of risk stratifying patients with AMI and should be used to accurately identify the risk of CI-AKI in AMI patients.

## Introduction

Acute renal injury (AKI), always associated with a poor prognosis, may arise from a variety of diseases (1). Acute myocardial infarction (AMI) is an important cause of AKI, due to its comorbidities, hemodynamic instability and the use of nephrotoxic drugs. Studies have shown that the incidence of AKI is between 11 and 26% in patients with AMI during hospitalization (2–6). The mortality rate among patients with AMI was found to be higher in the ones who developed AKI (1, 7, 8). Also, patients with AKI are more likely to develop long-term complications, including progression to chronic kidney disease, heart failure, recurrent myocardial infarction, and long-term mortality (9).

Early identification of patients with AMI, who are likely to develop contrast induced acute kidney injury (CI-AKI) after an invasive treatment, will alert us to start an early therapy (e.g., iso-osmolar contrast media, fluids, pre-procedural statin) to preserve the renal function. Certain risk biomarkers (10, 11) and predictive models (12, 13) were reported to be capable of predicting the incidence of AKI. However, their predictive efficiency needs further improvement. Moreover, the Precision Medicine Initiative requires physicians to avoid oversimplification of medical treatments and to take individual variability into account to improve the decision-making process.

Machine learning has a computational discipline that algorithms are formulated to model or recognize complex patterns or features, using large amounts of data. Previous studies have shown that some machine learning methods (e.g., Random Forest) are more accurate than the traditional logistic regression models in predicting disease prognosis (14–16). This provides us the inspiration to design this study.

The main purpose of this study is to compare the efficiencies of several popular machine learning techniques to predict CI-AKI in AMI patients. An additional objective is to show the clinical use of these machine learning methods.

## Results

### Baseline Characteristics

A total of 1,495 patients diagnosed with AMI were included in the study. The average age was 66.6 ± 13.9 years, and 71.2% of the sample were men. 66.4% of the participants had hypertension, 26.8% had diabetes, 49.8% patients had a history of smoking and 12.1% had a history of alcohol consumption. Among these patients, 63.1% were diagnosed with ST-segment elevation myocardial infarction (STEMI). During the procedure, 95.1% of the participants were given percutaneous coronary intervention (PCI) therapy (Table 1). Of all the enrolled patients, 226 (15.1%) developed CI-AKI after the procedure. We then divided the enrolled patients randomly into a training cohort (75%) and a validation cohort (25%). The baseline characteristics were compared in Supplementary Table 1. There were no significant differences between the two groups.

**Table 1:** Baseline characteristics for the study population.

**Characteristics**	**AMI patients (*n* = 1,495)**
Age, y	66.6 ± 13.9
Male, *n*%	1,065 (71.2%)
Systolic blood pressure, mmHg	132.4 ± 24.7
Diastolic blood pressure, mmHg	79.2 ± 16.6
Heart rate, beats per minute	80.9 ± 16.9
Body mass index, Kg/m^2^	23.7 ± 3.8
LVEF, %	49.9 ± 9.0
Smoking, *n*%	744 (49.8%)
Drinking, *n*%	181 (12.1%)
Hypertension, *n*%	993 (66.4%)
Diabetes, *n*%	400 (26.8%)
Killip class III or IV, *n*%	156 (10.4%)
STEMI, *n*%	943 (63.1%)
NSTEMI, *n*%	552 (36.9%)
**CI-AKI**
Yes	226 (15.1%)
No	1,269 (84.9%)
**Medications before procedures**, ***n*****%**	
Aspirin	1,445 (96.7%)
Clopidogrel	543 (36.3%)
Ticagrelor	952 (63.7%)
ACEI/ARB	882 (59%)
β-blocker	892 (59.7%)
Statins	1,337 (89.4%)
Low Molecular Heparin	1,464 (97.9%)
Tirofiban hydrochloride	728 (48.7%)
Digoxin	16 (1.1%)
Diuretics	287 (19.2%)
**Laboratory measurements at baseline**	
White blood cell, 10^9^/L	8.93 (6.96–11.48)
Neutrophil percentage, %	75.6 ± 10.8
Hemoglobin, g/L	133.5 ± 20.2
Serum creatinine, μmol/L	78.10 (64.60–96.75)
Uric acid, μmol/L	333 (274–404)
Serum albumin, g/L	37.8 ± 4.3
Blood glucose, mmol/L	6.90 (5.62–9.37)
Total triglycerides, mol/L	1.22 (0.90–1.78)
Total cholesterol, mmol/L	4.14 (3.52–4.81)
High-density lipoprotein cholesterol, mmol/L	1.10 (0.92–1.33)
Low-density lipoprotein cholesterol, mmol/L	2.39 (1.88–2.90)
Brain natriuretic peptide, pmol/L	1,031 (292–3,962)
Cardiac troponin I, ng/mL	1.76 (0.44–7.24)
Free triiodothyronine, pmol/L	4.0 ± 1.2
Free tetraiodothyronine, pmol/L	15.6 ± 3.3
**Procedural characteristics**, ***n*****%**	
Contrast volume > 100 ml	482 (32.2%)
Contrast exposure time > 60 min	200 (13.4%)
Use of IOCM	436 (29.2%)
Hydration therapy	344 (23%)
Pre-procedure hypotension	60 (4.0%)
CAG	1,495 (100%)
With adjunct PCI performed	1,421 (95.1%)
**Number of stents with each vessel**	
**Left main coronary artery**	
0	1,483 (99.2%)
1	12 (0.8%)
**Left anterior descending artery**	
0	753 (50.4%)
1	731 (48.9%)
≥2	11 (0.7%)
**Left circumflex artery**	
0	1,273 (85.2%)
1	221 (14.8%)
≥2	1 (0.1)
**Right coronary artery**	
0	1,038 (69.4%)
1	438 (29.3%)
≥2	19 (1.3%)

*LVEF, left ventricular ejection fraction; STEMI, ST segment elevation myocardial infarction; NSTEMI, non-ST segment elevation myocardial infarction; CI-AKI, acute kidney injury; IOCM, iso-osmolar contrast media; PCI, percutaneous coronary intervention; CAG, coronary angiography; Pre-procedure hypotension was defined as systolic blood pressure lower than 90 mmHg before procedure*.

### Logistic Regression Models

**Figure 1:**
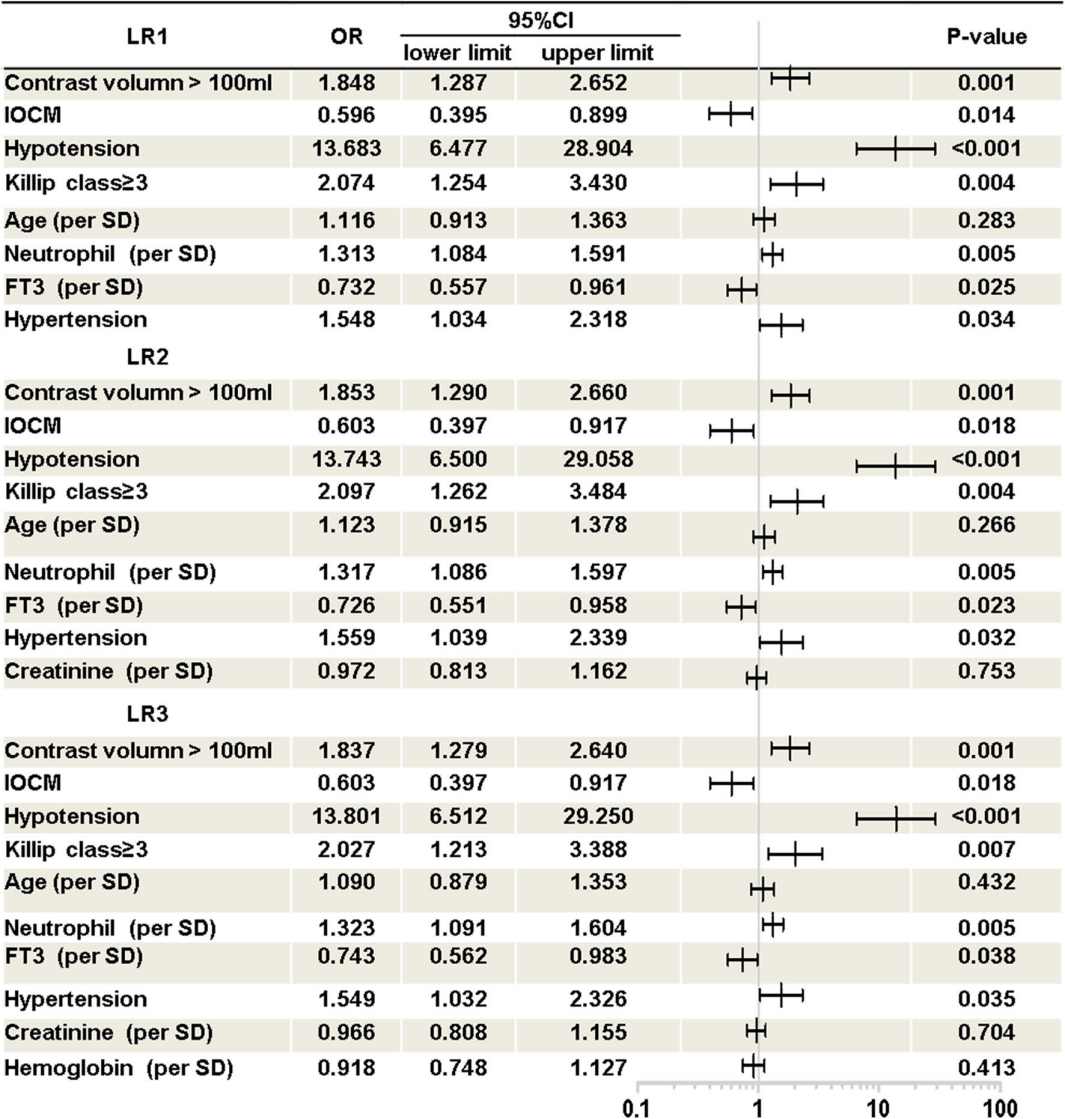
Logistic regression models. Presented is multivariate logistic regression analysis of CI-AKI in AMI patients. Three logistic regression model was developed. LR1, logistic regression model 1; LR2, logistic regression model 2; LR3, logistic regression model 3; OR, odds ratio; CI, confidence interval; IOCM, iso-osmolar contrast media; Hypotension, pre-procedure hypotension (systolic blood pressure below 90 mmHg before procedure); FT3, free triiodothyronine.

As shown in Figure 1, three Logistic models were developed. The following predictors were included in the models: contrast volume >100 ml, use of iso-osmolar contrast media (IOCM), hypotension, Killip class ≥ 3, age, neutrophil percentage, free triiodothyronine (FT3), hypertension, serum creatinine (SCr) and hemoglobin.

### Features Selection of the Machine Learning Models

**Figure 2:**
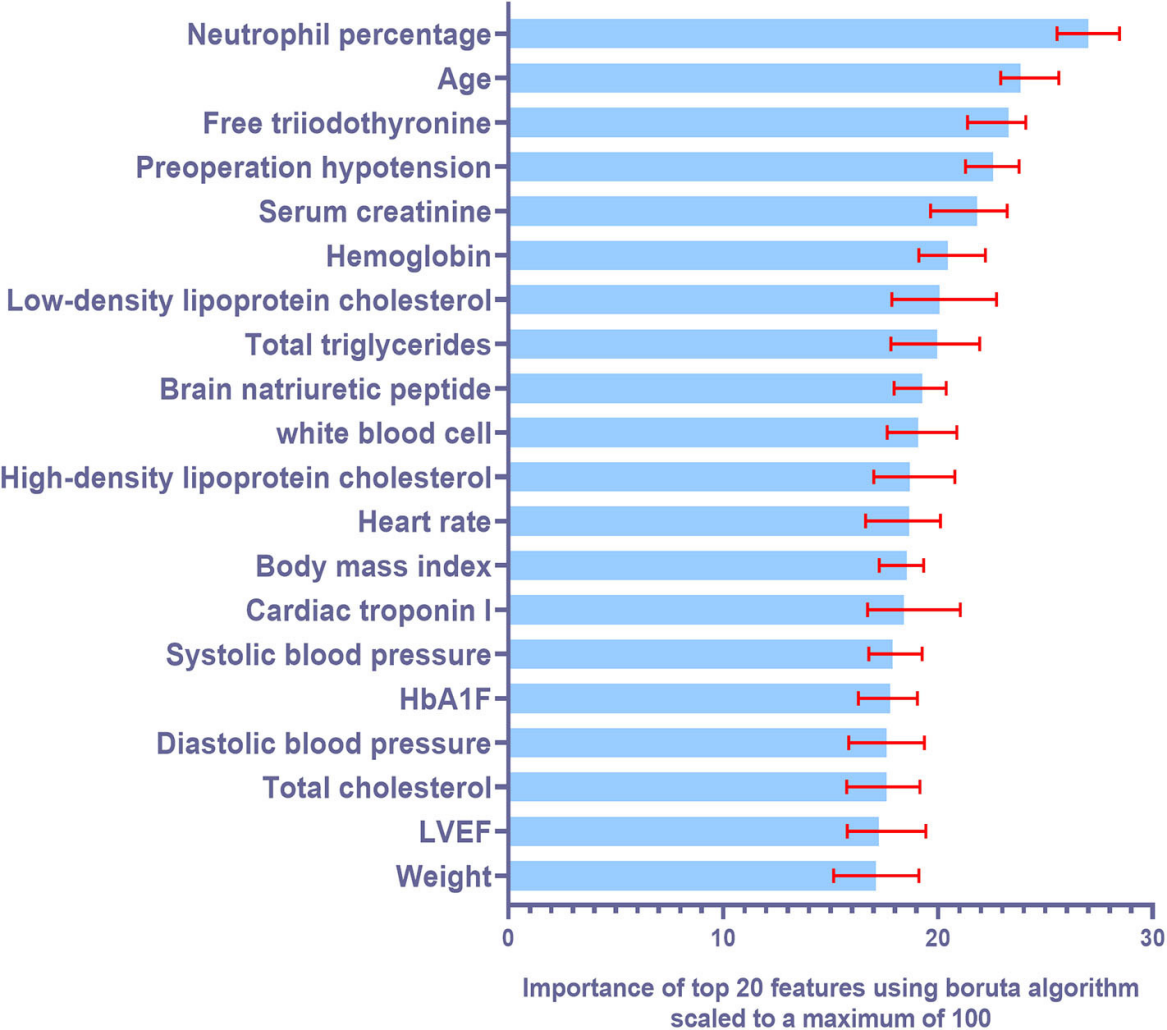
Summary of importance of the selected features according to Boruta algorithm. This figure shows the importance of top 20 ranked variables. The columns represent the medium importance of the feature and the error bars represent the maximum and minimal importance (scaled to a maximum value of 100).

Six machine learning models were constructed with features selected according to the training cohort. The models used were: decision tree (DT) model, support vector machine (SVM) model, random forest (RF) model, K-nearest neighbors (KNN) model, naive Bayes (NB) model, and gradient boosted machine (GBM) model. Ten-fold cross validation was also used while training the models. Figure 2 illustrates the top 20 features for CI-AKI using the Boruta Algorithm. The minimum and maximum importance of the top 20 features is also listed in Supplementary Table 2.

### Performances of all the Models in the Training Cohort

**Figure 3:**
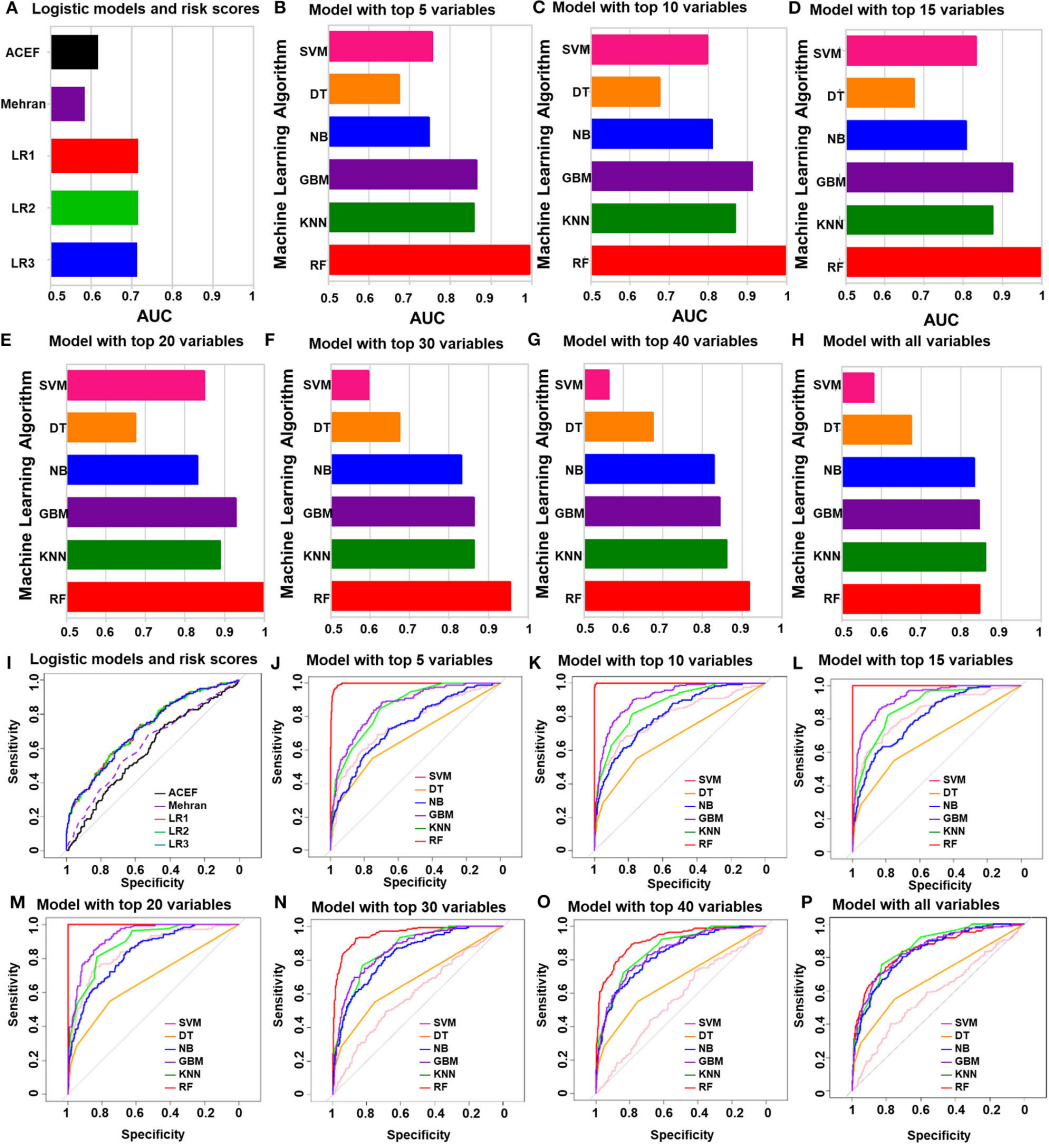
The performances of all of the models in the training group. **(A)** Logistic regression analysis and ACEF risk score and Mehran risk scores. **(B–H)** AUC of machine learning algorithm with top five variables **(B)**, top 10 variables **(C)**, top 15 variables **(D)**, top 20 variables **(E)**, top 30 variables **(F)**, top 40 variables **(G)** and all variables **(H)**. **(I)** ROC curve of logistic regression model and risk scores. **(J–P)** ROC curve of machine learning algorithm with top five variables **(J)**, top 10 variables **(K)**, top 15 variables **(L)**, top 20 variables **(M)**, top 30 variables **(N)**, top 40 variables **(O)** and all variables **(P)**.

The performances of all the models in the training cohort, including the logistic regression models and ACEF (age, creatinine, and ejection fraction) model, are shown in Figure 3. The ACEF model had an AUC of 0.59 (95% CI: 0.54–0.64) and Mehran risk score reached an AUC of 0.62 (95% CI: 0.57–0.67). The performance of the three logistic models in the training cohort was similar (Supplementary Table 3). The AUC of LR1, LR2, and LR3 was 0.72 (95% CI: 0.67–0.76), 0.72 (95% CI: 0.67–0.76), and 0.71 (95% CI: 0.67–0.76), respectively. All three logistic models performed better than the ACEF model or Mehran risk score. Except for the DT model, the rest of the five machine learning models performed better than the logistic models or ACEF models in the training cohort. The SVM model performed best when top 20 variables were added in the model (AUC of SVM-20 model: 0.85, 95% CI: 0.82–0.88). The DT model had the worst performance of all the machine learning models (AUC of DT-all variables: 0.68, 95% CI: 0.63–0.72). The NB performed well in the training cohort, and its AUCs increased when more variables were added (AUC of NB-all variables: 0.83, 95%CI: 0.80–0.87). The GBM model and KNN model performed better than the NB model (AUC of GBM-all variables: 0.85, 95% CI: 0.81–0.88; AUC of KNN-all variables: 0.86, 95% CI: 0.83–0.89). Among all the machine learning models in the training cohort, the RF model was the most accurate. The RF model achieved an AUC of 0.995 (95% CI: 0.993–0.998) with the top five variables included in the model. Receiver Operating Characteristic (ROC) curve of each model were showed in Figures 3I–P. The AUCs and 95% CI of each machine learning model are listed in Supplementary Table 4.

### Performances of all the Models in the Validation Cohort

**Figure 4:**
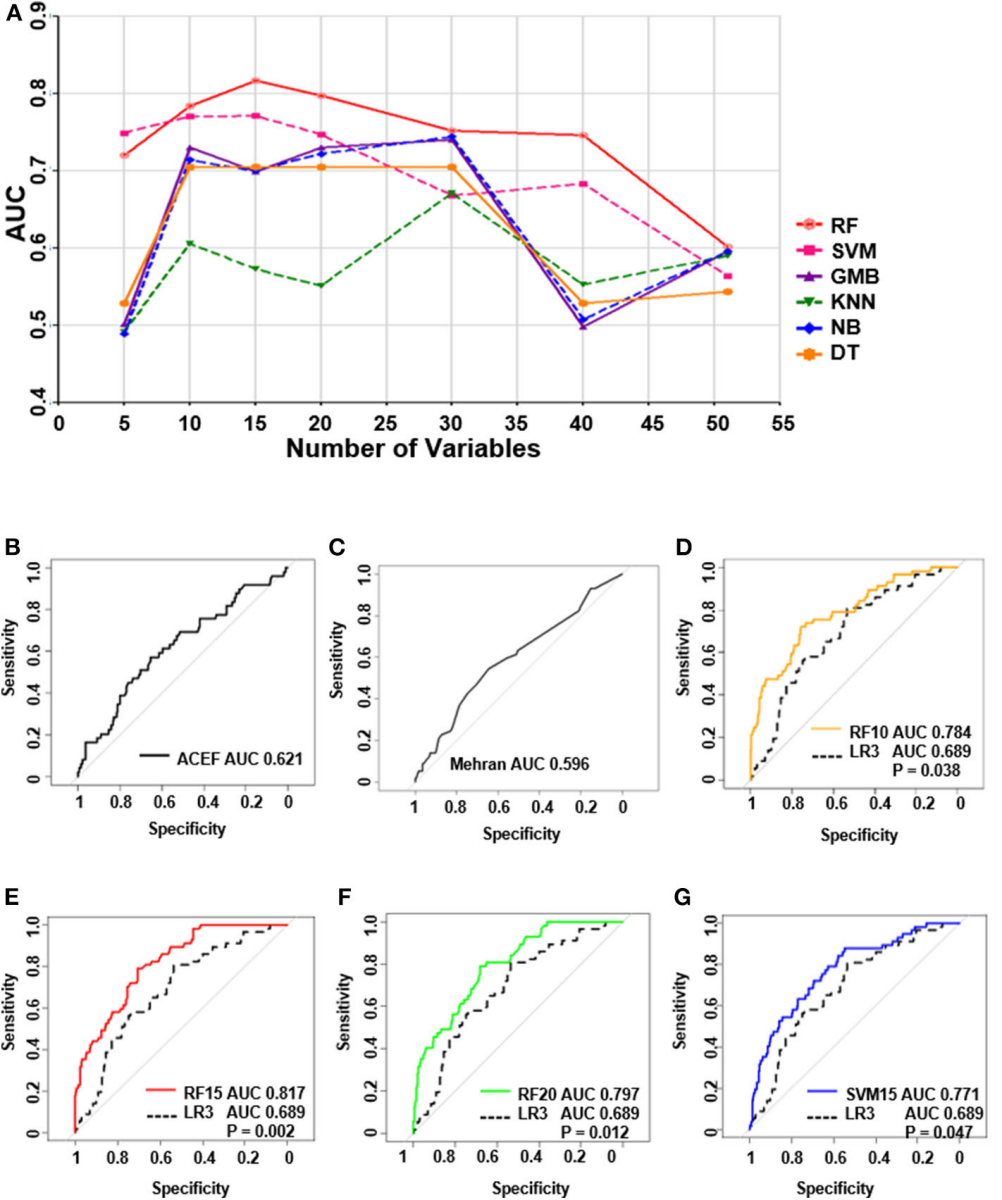
The performance of all the models in test group and receiver operating characteristic curve (ROC) of top 4 machine learning models, logistic regression and ACEF model. **(A)** The performances of all of the models in the validation group. **(B)** ROC curve of ACEF score. **(C)** ROC curve of Mehran risk score. **(D)** ROC analysis of (RF 10) Random forest model with top 10 variables. **(E)** ROC analysis of (RF 15) random forest model with top 15 variables. **(F)** ROC analysis of (RF 20) Random forest model with top 20 variables. **(G)** ROC analysis of (SVM 15) support vector machine model with top 15 variables.

Independent validation was conducted in a validation cohort of 373 cases. Of all the machine learning models developed, the RF model performed the best (Figure 4A). The top-four machine learning models were as follows: the RF-15 model, with an AUC of 0.82, (95% CI: 0.76–0.87); the RF-20 model, with an AUC of 0.80, (95% CI: 0.74–0.86); the RF-10 model, with an AUC of 0.78, (95% CI: 0.72–0.85); and the SVM-15 model, with an AUC of 0.77 (95% CI: 0.71–0.84). The ACEF model reached an AUC of 0.62 (95%CI: 0.53–0.71) and the AUC of Mehran risk score was 0.60, (95%CI: 0.51–0.68) (Supplementary Table 5). The performance of regression model LR3 was slightly better than LR1 and LR2 (LR3: AUC of LR3: 0.69, 95%CI: 0.62–0.76), (Supplementary Table 6).

### ROC Analysis of the Top Four Machine Learning Models and Logistic Model

ROC analysis in Figures 4B–G shows the underperformance of ACEF model and Mehran risk score. While all of the top four machine learning models performed significantly better than the LR3 model (all *P* < 0.05, bootstrap method, *n* = 2,000).

### Comparison of Top Four Machine Learning Models and Logistic Regression Model in the Validation Group

The Recall rate, F-1 score, and other metrics of the top four machine learning models and LR3 model were then compared (Table 2). The RF model with top 15 variables achieved a high recall rate of 71.9% and an accuracy of 73.5% in the validation group when the cut-off value was 0.5.

**Table 2:** Comparison of top-4 machine learning models and logistic regression model in validation group.

**Models**	**Observed CI-AKI (*****n*** **=** **57)**		**Observed non-CI-AKI (*****n*** **=** **316)**		**Cut-off**
	**True positive**	**False positive**	**False negative**	**True negative**	
RF10	42	15	91	225	0.50
	73.7%	26.3%	28.8%	71.2%	
RF15	41	16	83	233	0.50
	71.9%	28.1%	26.3%	73.7%	
RF20	37	20	84	232	0.50
	64.9%	35.1%	26.6%	73.4%	
SVM15	41	16	106	210	0.50
	71.9%	28.1%	33.5%	66.5%	
LR3	36	21	270	46	0.80
	63.2%	36.8%	85.4%	14.6%	
	**Precision**	**Recall**	**F1-score**	**Specificity**	**Accuracy**
RF10	31.6%	73.7%	44.2%	71.2%	71.6%
RF15	33.1%	71.9%	45.3%	73.7%	73.5%
RF20	30.6%	64.9%	41.6%	73.4%	72.1%
SVM15	27.9%	71.9%	40.2%	66.5%	67.3%
LR3	11.8%	63.2%	19.8%	14.6%	22.0%

*RF10, random forest model with top 10 variables; RF15, random forest model with top 15 variables; RF20, random forest model with top 20 variables; SVM15, support vector machine model with top 15 variables; LR3, logistic regression model 3*.

## Discussion

It is a large-scale study based on machine learning frameworks. The study used real-world data related to the patients with AMI to predict the possibility of CI-AKI in them. The results showed that machine learning methods are suitable for risk prediction in real-world research. First, the RF-based risk prediction method performed better than the logistic regression and Standard Risk methods. Second, the results suggest that the RF model, with the top 15 predictors, performed the best in CI-AKI prediction. Machine learning technology helps physicians to analyze a large amount of information and is crucial in medical practice optimization. Based on the current model, the computer-aided risk assessment does not need to manually calculate the score and predict the risk like the traditional risk score. The variables can be obtained from electronic medical record in our hospital. And the risk scores would be calculated automatically. So, it will be more convenient and rapid to apply for clinicians.

In our previous study (17), we found that Nomogram-based model gave better forecast accuracy results for CI-AKI in AMI patients, as compared to Mehran risk scores. Similar to the previous studies (17–19), our new data shows that machine learning models are superior to traditional logistic regression for developing predictive models. This finding makes sense because machine learning models are capable of learning complex discriminative features from large volumes of data without assumption of linearity. However, the discrepancy may be due to the features selected in our machine learning models. The RF model was built using the ensemble of decision trees. Thus, it will significantly boost predictive performance by reducing overfitting.

Boruta algorithm was used for feature selection in our machine learning models. After that, top five most powerful parameters, that is, neutrophil percentage, age of the patient, free triiodothyronine, hypotension and serum creatinine levels, were identified to be correlated with AKI. We found that the neutrophil percentage was the most important biomarker for predicting CI-AKI, suggesting that inflammatory response may play an important role in the occurrence of CI-AKI. Some studies suggest that age and creatinine levels are independent predictors of CI-AKI in AMI patients (20). Combined with our results, CI-AKI is more likely to occur in the elderly and in patients with poor basal renal function. Consistent with our study, it is also reported that free triiodothyronine had a negative association with CI-AKI in patients undergoing primary PCI therapy (6, 21). Preoperative hypotension may affect renal perfusion and lead to a higher risk of CI-AKI. These biomarkers are critical in improving the accuracy of our models. Moreover, the machine learning algorithm is helpful to combine the advantages of each biomarker, so as to obtain a more accurate model. There are several other advantages of using machine learning algorithms over traditional statistical modeling. As machine-learning algorithms consider all potential interactions and lack predefined assumptions, they are less likely to ignore unexpected predictor variables. Predictive models of machine-learning algorithm helped identify the risk of CI-AKI in patients with AMI, that otherwise would have gone unnoticed. Moreover, machine learning algorithms update themselves with the latest clinical data for higher accuracy. The prediction algorithms can be used to identify high-risk cases and help physicians optimize clinical decisions. In the near future, machine-learning algorithms can be expected to be used to develop an online risk calculator to assess CI-AKI risks in cardiac care units.

We recommend using machine learning models for the prediction of CI-AKI risk in AMI patients because machine learning models are superior to previously developed models at least in our study population. The use of the Random Forest algorithm significantly improved the predictive ability in comparison to traditional methods like regression analysis and risk scores. However, the addition of novel biomarkers and longitudinal data may still allow further refinement. We observed that the predictive models, which have readily available clinical data, can accurately identify the risk of CI-AKI after intervention in AMI patients. Prospective studies should be performed to demonstrate whether these models can identify the risk of CI-AKI in AMI patients at an earlier stage.

Our study has several limitations. Firstly, our study was performed in a single center with small sample size. The model has not been verified in the external validation queue. The future research should be carried on by expanding the sample size and optimizing the model to improve the prediction value of the model. Secondly, there are missing values in variables of different levels, which may bias the accuracy of prediction. Thirdly, newer CI-AKI biomarkers, such as GDF-15 (12, 13), cystatin C (22), and neutrophil gelatinase-associated lipocalin (NGAL) (23) were not included in the model because they are not generally detected at an early stage of the disease. Fourthly, serum creatinine was detected by the enzymatic method with creatininase coupled sarcosine oxidase. endogenous or exogenous substances may interfere with the determination of serum creatinine compared with LC-MS/MS method. Despite these limitations, our models achieved higher accuracy and better performance than logistic regression models and ACEF models, indicating that the advantages of this study, specifically its novel methodology, outweigh its limitations. It can be expected that the models will be validated in other cohorts in the future.

In conclusion, our study shows that machine learning will help identify patients with the highest risk of CI-AKI in patients with AMI. In addition, it will identify the most important factors associated with increased risk of CI-AKI. However, the clinical management to reduce the risk of CI-AKI was not addressed. In the future, prospective studies will explore whether we can use machine learning models to stratify at-risk patients and target higher-level care for high-risk patients.

## Methods

### Study Population and Study Design

**Figure 5:**
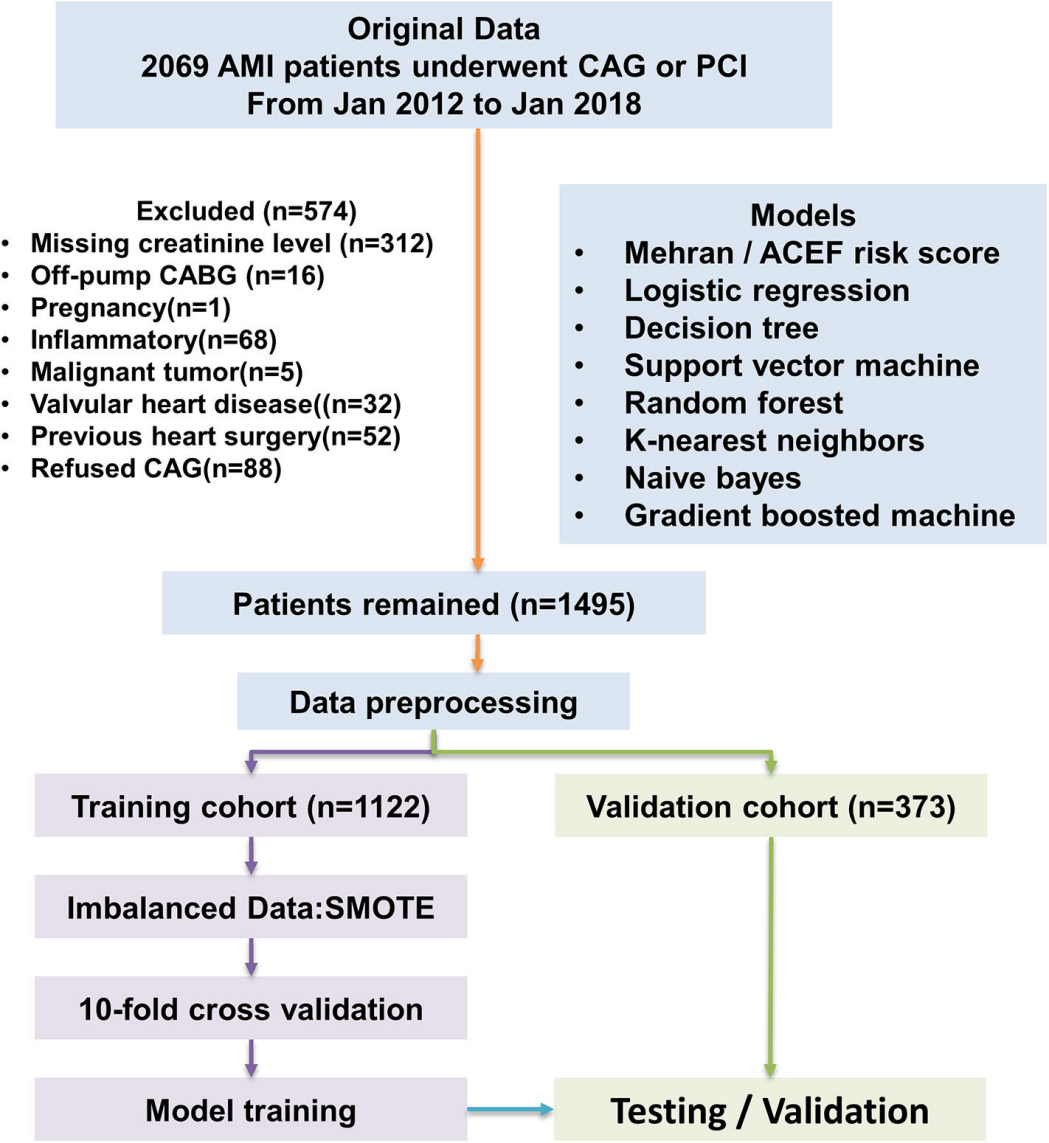
Flow diagram of study. Model development performed with 1,122 AMI patients. ACEF model, Mehran risk score, logistic regression model, and machine learning algorithms were tested in the validation cohort. AMI, acute myocardial infraction; CAG, coronary angiography.

This is a retrospective, observational cohort study. The study was conducted in Changzhou No.2 People's Hospital of Nanjing Medical University. The study population included adult patients with clinically diagnosed acute myocardial infarction (AMI) from January 2012 to January 2018. All enrolled patients provided written informed consent. All AMI patients enrolled had underwent coronary angiography. Percutaneous interventional therapy (PCI) was performed according to Chinese Guidelines for Percutaneous Coronary Intervention (2016). Briefly, PCI should be based on the degree of coronary artery stenosis. When the diameter of the lesion is more than 80%, it can be directly intervened; when the diameter of the lesion is <80%, it is suggested to intervene only those lesions with corresponding ischemic evidence or with FFR ≤ 0.8. The type and volume of contrast medium, operating time and severity of coronary lesion were recorded. The pharmacological treatments of each patient were performed according to the Guidelines for the Treatment of Coronary Heart Disease in China (2016), including anticoagulant, antiplatelet and lipid-lowering therapy. Socio-demographic data, pre-procedural vital signs, investigations was also collected from the electronic medical records system. The definition of AMI was according to “the Third Universal Definition of Myocardial Infarction from the Joint ESC/ACCF/AHA/WHF Task Force” (24). All enrolled patients were randomly divided into a train cohort (75%) and a validation cohort (25%). Our study flow chart is shown in Figure 5.

### Study Endpoint

The study endpoint was CI-AKI after the procedure (administration of contrast media). According to the serum creatinine (SCr)-based criteria provided by the Kidney Disease Improving Global Outcomes (KDIGO) (25), CI-AKI is defined as an absolute increase in serum creatinine at 48 h of procedure by ≥ 0.3 mg/dl or an increase of more than or equal to 150% from its baseline value within the prior 7 days, or urine volume <0.5 ml/kg/h for 6 h. Creatinine was detected by the enzymatic method with creatininase coupled sarcosine oxidase.

### Pre-processing of the Datasets

Because the models required a complete dataset, the missing data of each remaining measurement was estimated using the K-nearest neighbor method (26). The variables were standardized after the K-nearest neighbor imputation method was used. Variables that were missing in more than 50% of the samples were removed (e.g., glucose, urine acid, and albumin). Next, ambiguous measurements that did not carry specific meaning (e.g., a variable named “history of the smoking” not specific to a length of time) were removed. Finally, redundant variables derived only from other measured variables were removed (e.g., estimated Glomerular Filtration Rate is derived from serum creatinine, sex, age, and weight and is therefore redundant).

### ACEF Risk Score and Mehran Risk Score

The ACEF risk score was calculated using three variables (age, creatinine, ejection fraction), according to Ranucci et al. (27). The formula of ACEF score is age/ejection fraction (%) + 1 (if serum creatinine ≥2.0 mg/dl). (Consider using: The ACEF Score was calculated using Age/Ejection fraction (%) + 1 (if SCr is ≥ 2.0 mg/dl). Mehran risk score (28) was also calculated in each patient in training group and validation group.

### Development of Regression Models

Three predictive logistic regression models were developed for predicting CI-AKI after the procedure in the training cohort. Univariate analysis of the training cohort was conducted and the variables with *P* < 0.1 were included in the multivariate analysis model. Logistic regression model 1 (LR1) was then developed. Creatinine and hemoglobin were included to form logistic model 2 (LR2) and logistic model 3 (LR3).

### Development of Machine Learning Models

For the **Decision Tree (DT) model** (29, 30), the sample data was partitioned, by splitting the variables at discrete cut-points, and presented graphically in the form of a tree. As Decision Trees often have suboptimal predictive accuracy, several methods were used to combine the multiple trees together. First, **a Random Forest (RF) model** was applied (31, 32). Random Forest operates by constructing modified bagged trees that only allow a random sample of the predictor variables to be considered at each split of each tree. **Gradient Boosting Machine (GBM)** is a forward learning ensemble method (33). GBM produces a prediction model, usually a decision tree, in the form of an ensemble of weak prediction models. GBM trains models in a stage-wise manner, as do other boosting methods, and it generalizes those weak prediction models by optimizing any arbitrary differentiable loss function.

Support Vector Machines (SVM), a supervised machine learning method (34), was used for classification or regression by constructing a hyperplane or set of hyperplanes. It is to be noted that, the larger the margin, the lower will be the generalization error of the classifier. In our study, the hyperplanes, constructed in SVM, achieved a good separation, with the largest distance to the nearest training-data point of any class (functional margin).

The **K-nearest Neighbor's algorithm (k-NN)** (35), a non-parametric method, was used to deal with classification and regression. The input consists of the k closest training examples in the feature space, while the output depends on whether k-NN is used for classification or regression. The output is a class membership in k-NN classification. The sample belongs to a category of feature space that has a majority of the similar type of k samples. The object is assigned to the class of that single-nearest neighbor when *k* = 1.

The **Naive Bayes** classifier, based on Baye's theorem, is a simple “probabilistic classifier,” with a strong assumption of independence. The assumption made here is that the presence of one feature has no effect on the other, that is, features are independent (36).

### Performance Evaluation

We assessed the performance of each prediction model using area under the receiver operating characteristic curve (AUROC). For the final models, we calculated the optimal cut-off value. Moreover, a confusion matrix was calculated according to the cut-off value. We also compared the results of the precision, recall (37), F1-score (37), specificity, and accuracy of final model in each of the test groups. The formula of the above metrics is as follows: precision = TP/(TP+FP); recall = TP/(TP+FN); specificity TN/(TN+FP); accuracy (TP+TN)/(TP+FP+TN+FN); F1-score = P*R/2(P+R). TP represented True Positive rate, FP represented False Positive rate, TN represented True Negative rate, FN represented False Negative rate. In the formula of F1-Score, P represented Precision and R represented Recall rate.

### Statistical Analysis

Mean ± standard deviation (SD), median and 25th−75th percentiles were used to represent continuous variables. The categorical variable was represented by the absolute value (percent). Student *t* test, Wilcoxon rank sum test, and chi-square test were used to compare the demographic and clinical characteristics of the CI-AKI patients and non-CI-AKI patients. The enrolled patients were divided randomly into two sets: a training set for model development with 75% of the patients and a validation set for model validation with 25% of the patients. Ten-fold cross-validation was used while training the machine learning models. In ten-Fold cross-validation, the original samples are randomly divided into ten subsamples of equal size, in which one subsample is used as the validation data and the remaining nine subsamples are used as the training data. The advantages of ten-Fold cross-validation are that entire data of observation is used for training and validation, and each observation gets validated only once. Synthetic Minority Oversampling Technique (SMOTE) (38) was used to deal with an imbalanced dataset. Our dataset consisted of 1,495 patients, with heterogeneous samples of CI-AKI and non-CI-AKI patients. CI-AKI patients represented only 15.1% of the whole sample, while non-CI-AKI patients represented 84.9%. The variance between these two classes is considerably large and may lead to a lower prediction accuracy for the predictive models. The SMOTE technique is a powerful technique to tackle imbalanced data distribution. The SMOTE method was based on two techniques: random under-sampling and the synthetic minority over-sampling technique. After the dataset were handled with SMOTE method, we developed ACEF model, logistic regression model and other six machine-learning models. The Boruta algorithm was used for feature selection in the machine learning models (39). The Boruta package relies on an RF classification algorithm, which provides an intrinsic measure of the importance of each feature, called the Z score. This score is not a direct statistical measure to estimate the significance of the feature.

Then, the six types of machine learning models with all the variables (top 40, 30, 20, 15, 10, and 5 variables), were separately developed. We calculated the AUC of each model and evaluated the performance of all the models in the training cohort. A validation cohort was used to internally validate the models. The AUC and 95% CI were calculated and compared for each model. The Bootstrap method was used while comparing the AUCs of each model. Moreover, the Precision, Recall, F1-score, Specificity and Accuracy of each final model in the validation cohort were also compared. All analyses were performed using IBM SPSS Statistics (version 22) and RStudio (version 1.0.153). Packages of “foreign,” “caret,” “Boruta,” “DMwR,” “Random Forest,” and” pROC” were used to process the datasets. A *p*-value of < 0.05 was considered statistically significant.

## Data Availability Statement

The raw data supporting the conclusions of this article will be made available by the authors, without undue reservation.

## Ethics Statement

The studies involving human participants were reviewed and approved by Changzhou No.2 People's Hospital Review Board [number (2018) KY005-01]. The patients/participants provided their written informed consent to participate in this study.

## Author Contributions

LS, QW, and FZ conceived the experiments. WZ, XC, YJ, JJ, NL, YX, YZ, and ZS conducted the experiments. FZ, JJ, LS, and QW analyzed the results. All authors reviewed the manuscript.

## Conflict of Interest

The authors declare that the research was conducted in the absence of any commercial or financial relationships that could be construed as a potential conflict of interest.
