# Supplementary material for: Machine Learning to Predict Contrast-Induced Acute Kidney Injury in Patients With Acute Myocardial Infarction
Source: Front Med (Lausanne). 2020 Nov 13;7:592007. doi: 10.3389/fmed.2020.592007 (PMC7691423; doi:10.3389/fmed.2020.592007)
Supplement: Supplementary file 1 [file Data_Sheet_1.PDF]

Machine Learning to Predict Contrast-induced Acute Kidney Injury in Patients with Acute Myocardial Infarction

Ling Sun[1,a], Wenwu Zhu[2,a], Xin Chen[1,a], Jianguang Jiang[1], Yuan Ji[1], Nan Liu[3], Yajing Xu[1], Yi Zhuang[1], Zhiqin Sun[4], Qingjie Wang[1,#],Fengxiang Zhang[2,#]

[1]Department of Cardiology, The Affiliated Changzhou No. 2 People's Hospital of Nanjing Medical University, Changzhou, China, 213003

[2]Section of Pacing and Electrophysiology, Division of Cardiology, the First Affiliated Hospital of Nanjing Medical University, Nanjing, China, 210009

[3]Department of DSA, The Affiliated Changzhou No. 2 People's Hospital of Nanjing Medical University, Changzhou, China, 213003

[4]School of Clinical Medicine, The Affiliated Changzhou No. 2 People's Hospital of Nanjing Medical University, Changzhou, China, 213003

[a]These authors contributed equally to this study

[#]Address for correspondence:

Qingjie Wang, MD, phD. Department of Cardiology, the Affiliated Changzhou No.2 People's Hospital of Nanjing Medical University, 29 Xinglong Alley, Changzhou, 213003, PR China. Email: wang-qingjie@hotmail.com, Tel: 86-519-81087104

Fengxiang Zhang, MD. Section of Pacing and Electrophysiology, Division of Cardiology. The First Affiliated Hospital of Nanjing Medical University. Guangzhou Road 300, Nanjing, 210029, PR China. E-mail address: njzfx6@njmu.edu.cn, Tel/Fax: 86-25-83717168.

eTABLE 1. Baseline Characteristics for the Study Population

**Characteristics**	**Training group (n=1122)**	**Validation group (n=373)**	**P value**
Age, y	66.9 ± 13.9	65.7 ± 13.8	0.149
Male, n%	794 (70.8%)	271 (72.7%)	0.485
Systolic blood pressure, mmHg	132.2 ± 24.2	132.8 ± 26.2	0.714
Diastolic blood pressure, mmHg	79.1 ± 16.4	79.7 ± 17.1	0.509
Heart rate, beats per minute	80.7 ± 16.8	81.5 ± 17.0	0.414
Body mass index, Kg/m2	23.6 ± 3.7	24.0 ± 4.0	0.224
LVEF, %	49.9 ± 9.1	50.0 ± 8.5	0.868
Smoking, n%	553 (49.3%)	191 (51.2%)	0.521
Drinking, n%	142 (12.7%)	39 (10.5%)	0.259
Hypertension, n%	749 (66.8%)	244 (65.4%)	0.635
Diabetes, n%	308 (27.5%)	92 (24.7%)	0.292
Killip class Ⅲ or Ⅳ, n%	113 (10.1%)	43 (11.5%)	0.425
STEMI, n%	712 (63.5%)	231 (61.9%)	0.596
NSTEMI, n%	410 (36.5%)	142 (38.1%)	0.596
CI-AKI			0.918
Yes	169 (15.1%)	57 (15.3%)	
No	953 (84.9%)	316 (84.7%)	
Contrast volume ＞ 100ml	353 (31.9%)	124 (33.2%)	0.632
Contrast exposure time ＞ 60min	154 (13.7%)	46 (12.3%)	0.494
Use of IOCM	340 (30.3%)	96 (25.7%)	0.093
Hydration therapy	268 (23.9%)	76 (20.4%)	0.163
Preprocedural hypotension	40 (3.6%)	20 (5.4%)	0.126
CAG only	56 (5.0%)	18 (4.8%)	0.899
With adjunct PCI performed	1066 (95.0%)	355 (95.2%)	0.899

LVEF= left ventricular ejection fraction; STEMI=ST segment elevation myocardial infarction; NSTEMI=non-ST segment elevation myocardial infarction; CI-AKI=acute kidney injury; IOCM=iso-osmolar contrast media; PCI=percutaneous coronary intervention; CAG=coronary angiography; Preprocedural hypotension was defined as systolic blood pressure lower than 90mmHg before procedure.

eTable2. Importance of top 20 features using Boruta algorithm					
Rank	features	mean Importance	median Importance	minimum Importance	maximum Importance
1	Neutrophil percentage	27.065	27.010	25.554	28.453
2	Age	23.932	23.842	22.931	25.630
3	Free triiodothyronine	23.100	23.283	21.380	24.089
4	Preoperational hypotension	22.551	22.572	21.282	23.780
5	Serum creatinine	21.642	21.832	19.664	23.228
6	Low-density lipoprotein cholesterol	20.192	20.072	17.853	22.727
7	Hemoglobin	20.503	20.484	19.112	22.213
8	Total triglycerides	19.977	19.961	17.817	21.947
9	Cardiac troponin I	18.466	18.428	16.724	21.047
10	white blood cell	19.091	19.071	17.643	20.878
11	High-density lipoprotein cholesterol	18.826	18.685	17.015	20.792
12	Brain natriuretic peptide	19.451	19.277	17.961	20.391
13	Heart rate	18.484	18.653	16.633	20.119
14	LVEF	17.343	17.256	15.783	19.437
15	Diastolic blood pressure	17.517	17.615	15.843	19.367
16	Body mass index	18.527	18.548	17.270	19.341
17	Systolic blood pressure	17.853	17.907	16.782	19.266
18	Total cholesterol	17.573	17.615	15.754	19.164
19	Weight	17.226	17.104	15.150	19.122
20	HbA1F	17.714	17.777	16.299	19.044

eTABLE 3. AUC of logistic and ACEF models for predicting AKI in training group				
**Variables**	**AUC**	**95%CI**		**P value**
		**lower limit**	**upper limit**	
Logistic regression model 1（LR1）	0.716	0.673	0.758	<0.001
Logistic regression model 2（LR2）	0.716	0.674	0.759	<0.001
Logistic regression model 3（LR3）	0.714	0.672	0.757	<0.001
ACEF model (n=979)	0.585	0.536	0.635	0.001
Mehran risk score	0.618	0.571	0.665	<0.001

eTABLE 4. AUC of each machine learning models for predicting AKI in training group				
**Variables**	**AUC**	**95%CI**		**P value**
		**lower limit**	**upper limit**	
GBM with top 5 variables	0.865	0.836	0.894	<0.001
NB with top 5 variables	0.747	0.706	0.788	<0.001
KNN with top 5 variables	0.858	0.831	0.885	<0.001
RF with top 5 variables	0.995	0.993	0.998	<0.001
DT with top 5 variables	0.675	0.626	0.724	<0.001
SVM with top 5 variables	0.756	0.714	0.799	<0.001
GBM with top 10 variables	0.912	0.891	0.934	<0.001
NB with top 10 variables	0.810	0.776	0.845	<0.001
KNN with top 10 variables	0.869	0.841	0.897	<0.001
RF with top 10 variables	1.000	1.000	1.000	<0.001
DT with top 10 variables	0.675	0.626	0.724	<0.001
SVM with top 10 variables	0.797	0.755	0.838	<0.001
GBM with top 15 variables	0.925	0.905	0.944	<0.001
NB with top 15 variables	0.807	0.773	0.841	<0.001
KNN with top 15 variables	0.875	0.849	0.901	<0.001
RF with top 15 variables	1.000	1.000	1.000	<0.001
DT with top 15 variables	0.675	0.626	0.724	<0.001
SVM with top 15 variables	0.832	0.796	0.867	<0.001
GBM with top 20 variables	0.928	0.911	0.945	<0.001
NB with top 20 variables	0.831	0.799	0.862	<0.001
KNN with top 20 variables	0.889	0.865	0.913	<0.001
RF with top 20 variables	1.000	1.000	1.000	<0.001
DT with top 20 variables	0.675	0.626	0.724	<0.001
SVM with top 20 variables	0.849	0.817	0.881	<0.001
GBM with top 30 variables	0.863	0.835	0.891	<0.001
NB with top 30 variables	0.831	0.799	0.863	<0.001
KNN with top 30 variables	0.863	0.836	0.890	<0.001
RF with top 30 variables	0.955	0.939	0.971	<0.001
DT with top 30 variables	0.675	0.626	0.724	<0.001
SVM with top 30 variables	0.596	0.549	0.644	<0.001
GBM with top 40 variables	0.843	0.811	0.874	<0.001
NB with top 40 variables	0.829	0.797	0.862	<0.001
KNN with top 40 variables	0.860	0.832	0.888	<0.001
RF with top 40 variables	0.917	0.894	0.940	<0.001
DT with top 40 variables	0.675	0.626	0.724	<0.001
SVM with top 40 variables	0.563	0.516	0.610	0.009
GBM with all variables	0.845	0.813	0.878	<0.001
NB with all variables	0.833	0.801	0.865	<0.001
KNN with all variables	0.860	0.832	0.887	<0.001
RF with all variables	0.846	0.812	0.881	<0.001

DT with all variables	0.675	0.626	0.724	<0.001
SVM with all variables	0.580	0.531	0.629	0.001
DT= Decision tree; SVM= Support vector machine; RF= Random forest; KNN= K-nearest neighbors; NB= naive bayes; GBM= gradient boosted machine.				

eTABLE 5. AUC of each machine learning models for predicting AKI in validation group				
Variables	AUC	95%CI		P value
		lower limit	upper limit	
GBM with top 5 variables	0.700	0.623	0.777	<0.001
NB with top 5 variables	0.756	0.685	0.827	<0.001
KNN with top 5 variables	0.636	0.556	0.717	0.001
RF with top 5 variables	0.720	0.646	0.795	<0.001
DT with top 5 variables	0.705	0.622	0.788	<0.001
SVM with top 5 variables	0.749	0.678	0.821	<0.001
GBM with top 10 variables	0.730	0.657	0.803	<0.001
NB with top 10 variables	0.714	0.637	0.791	<0.001
KNN with top 10 variables	0.606	0.529	0.684	0.011
RF with top 10 variables	0.784	0.718	0.850	<0.001
DT with top 10 variables	0.705	0.622	0.788	<0.001
SVM with top 10 variables	0.770	0.696	0.845	<0.001
GBM with top 15 variables	0.740	0.669	0.810	<0.001
NB with top 15 variables	0.744	0.675	0.814	<0.001
KNN with top 15 variables	0.671	0.596	0.745	<0.001
RF with top 15 variables	0.817	0.763	0.870	<0.001
DT with top 15 variables	0.705	0.622	0.788	<0.001
SVM with top 15 variables	0.771	0.705	0.836	<0.001
GBM with top 20 variables	0.730	0.660	0.800	<0.001
NB with top 20 variables	0.722	0.653	0.791	<0.001
KNN with top 20 variables	0.551	0.473	0.629	0.217
RF with top 20 variables	0.797	0.740	0.855	<0.001
DT with top 20 variables	0.705	0.622	0.788	<0.001
SVM with top 20 variables	0.747	0.676	0.817	<0.001
GBM with top 30 variables	0.699	0.625	0.773	<0.001
NB with top 30 variables	0.700	0.629	0.770	<0.001
KNN with top 30 variables	0.573	0.492	0.653	0.079
RF with top 30 variables	0.752	0.687	0.817	<0.001
DT with top 30 variables	0.705	0.622	0.788	<0.001
SVM with top 30 variables	0.668	0.597	0.739	<0.001
GBM with top 40 variables	0.696	0.624	0.769	<0.001
NB with top 40 variables	0.713	0.645	0.782	<0.001
KNN with top 40 variables	0.586	0.505	0.667	0.039
RF with top 40 variables	0.746	0.682	0.809	<0.001
DT with top 40 variables	0.705	0.622	0.788	<0.001
SVM with top 40 variables	0.683	0.614	0.752	<0.001
GBM with all variables	0.596	0.522	0.669	0.022
NB with all variables	0.596	0.519	0.672	0.021
KNN with all variables	0.590	0.515	0.665	0.031
RF with all variables	0.602	0.525	0.680	0.014

DT with all variables	0.544	0.461	0.627	0.286
SVM with all variables	0.564	0.486	0.643	0.124

DT= Decision tree; SVM= Support vector machine; RF= Random forest; KNN= K-nearest neighbors; NB= naive bayes; GBM= gradient boosted machine.

eTABLE 6. AUC of logistic and ACEF models for predicting AKI in validation group				
**Variables**	**AUC**	**95%CI**		**P value**
		**lower limit**	**upper limit**	
Logistic regression model 1（LR1）	0.681	0.610	0.753	<0.001
Logistic regression model 2（LR2）	0.681	0.610	0.752	<0.001
Logistic regression model 3（LR3）	0.689	0.618	0.759	<0.001
ACEF model (n=333)	0.621	0.534	0.708	0.007
Mehran risk score	0.596	0.514	0.679	0.021
